# Diverging trajectories of trust in healthcare and on-line information seeking: what’s next with LLMs

**DOI:** 10.1038/s41746-026-02408-9

**Published:** 2026-01-31

**Authors:** Ryan A. Heumann, Steven R. Steinhubl

**Affiliations:** 1 Purdue University, Lafayette, IN USA; 2 Indiana University School of Medicine, Indianapolis, IN USA

**Keywords:** Business and industry, Health care, Mathematics and computing

## Abstract

As technology evolves, so does the way people engage with it—especially when it comes to health information. Over the past 25 years, the explosive growth in internet use has been paralleled by an equally rapid increase in individuals seeking health information online. During this same period, public trust in the United States healthcare system has steadily eroded. While a direct causal link is unlikely, these trends are interconnected. The primary drivers of declining trust – cost and limited accessibility – have pushed many people to seek alternatives. Yet, health systems have historically paid little attention to the underlying motivations driving people to seek health information independently. The rapid emergence of large language models (LLMs), which provide unprecedented access to personalized and context-aware health information, signals a profound shift in this landscape. As LLMs become increasingly integrated into everyday information-seeking behaviors, they may further supplant clinicians as the initial point of contact for health-related information and guidance. Rather than viewing this long-standing, but rapidly accelerating shift towards patients seeking health guidance online as peripheral to traditional care, patient-centric health systems have an opportunity to harness it. By acknowledging their value to patients and integrating the ways people use online resources to inform their health decisions, systems of care can cultivate greater trust, strengthen health literacy, and deepen patient engagement. Informed by past missteps, the path forward for healthcare rests not in competing with these technologies, but in collaborating with the tools people are already choosing to guide their health.

## Introduction

The implementation of new technology in medicine is far from a straightforward process. Even the most promising of developments can introduce controversy and unintended consequences. In fact, the invention of the stethoscope was met with resistance from physicians who disapproved of the tool because it interfered with the physical relationship between them and their patients^1^. However, over time, the relationship evolved to include this new piece of technology and today the stethoscope is central to the identity of a clinician^2^. Over the past three and a half decades, another transformative technology - the internet - has emerged. Since 1991, global internet usage has skyrocketed from less than 1% of the population (4.2 million users) to more than two-thirds of the world—over 5 billion people^3^. Additionally, though not originally designed for healthcare, it is now used over a billion times a day for health-related purposes^4^.

Historically, the physician-patient relationship is rooted in a paternalistic framework, where patients who were largely unable to access relevant health information were expected to rely on physicians to share the information they felt necessary and make decisions for them^5^. Hippocrates himself argued that physicians hold the responsibility of deciding how much shared information is in the patient’s best interest^1^. Today, with the enormous accessibility of medical information available online, patients are no longer dependent on this paternalistic dynamic, and instead can operate as an active participant in the triadic one between them, their physician, and what has become known as “Doctor Google”^5,6^. Thus, as the popularity of internet use for health information seeking continues to grow, the relationship between patient and provider continues to evolve.

Trust is fundamental to healthcare, yet in the US at least, it has been steadily declining over the 6 decades it has been measured^7^. While there is no way of knowing what role, if any, the rise of online health information plays in this, it is important to understand how changes in patients’ trust - both in their individual physician and the healthcare system as a whole and the allure of online health information share several commonalities.

The investigation of this link becomes even more urgent as we enter a new era of digital health information. A tidal wave of patient-facing generative artificial intelligence (AI) tools - particularly conversational agents powered by large language models (LLMs) – is rapidly reshaping how people access and engage with health information. Twenty-five years ago, in the early days of the internet, Jerome Kassirer, the former Editor of the New England Journal of Medicine, issued a prescient warning about the emerging digital landscape in medicine: *“There are substantial risks of deep involvement by physicians in transforming medicine, yet there are also grave risks of opting out. The question is whether physicians will participate themselves or leave it as a medium for others to control. Should they decide not to participate, they risk seeing the importance of their role as educators for their patients diminish at the same time that their patients risk being misled by bad health information”*^8^. His words are even more relevant today. Just as the stethoscope once redefined the physician-patient relationship, the rise of AI in healthcare demands a similar evolution.

## Growth of Online Health Information Seeking

**Fig. 1:**
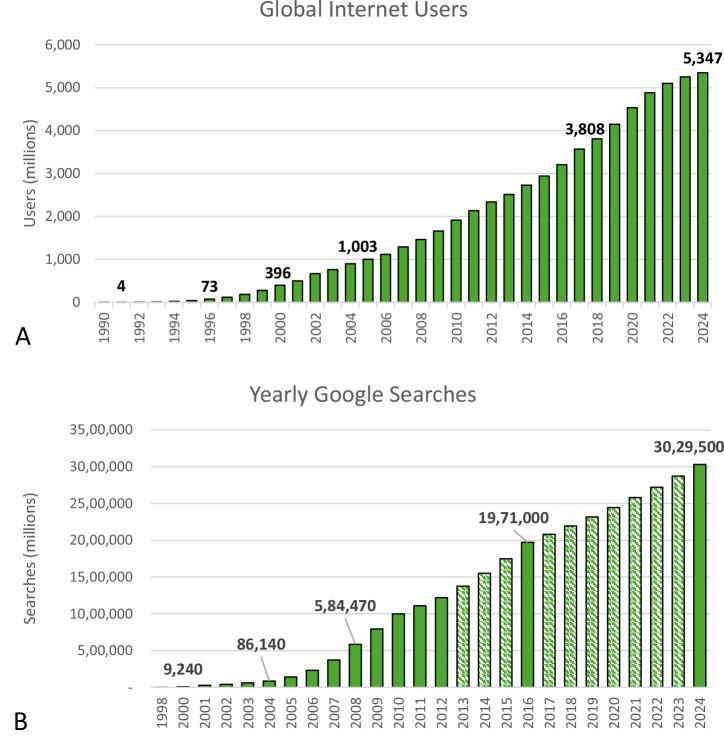
Change in internet use from 1990-2024. **A** Global internet users growth from 1990 to 2024. (Data from reference #4) and (**B**) Annual number of searches on Google from 1998 through 2024. Dashed bars represent results from exponential interpolation. (Data from references #13 and #14).

The internet era known as “Web 1.0” began in the mid-1990s as basic web browsers were developed that allowed the roughly 50 million users to access e-mail and news in real-time^9^. The Web 2.0 era, characterized by user-generated content and social connectivity, emerged in 2000^9^. Around this time, the number of internet users surpassed 400 million and the capabilities of Web 2.0 allowed increased interaction between them^9^. Over the next several decades, the number of internet users grew exponentially – to over one billion in 2005 and over five billion in 2024^3^. (Fig. 1A) Notably, as the number of internet users has grown, so has the proportion of those who have begun to use it to find health information.

What exactly this health information consists of varies from user to user. Before a visit to a physician, a person might search online for a better understanding of their symptoms, possible diagnoses, logistics like what provider to see and where, or even for reassurance that would allow them to avoid spending time and money seeing a physician in the first place^6,10^. After a physician visit, a patient might search for further clarification on information provided to them or for perspectives and advice from other people who have received similar diagnoses^6^. No matter what the motivation, individuals regularly cite the affordability, convenience, and inexhaustibility of online sources of health information^6^.

The popularity and growing demand for online health information is well established. In 2017, 74% of US adults stated that they go online first when they have a health-related question – an increase from 61% in 2008^11^. In 2022, a separate survey found that 85% of US internet users had searched online for health or medical information in the past year^12^. The same year, a third of internet users between the ages of 16 and 64 years cited researching health issues as a main motivation for using the internet, with over a quarter checking health symptoms online weekly^13^. In fact, it is estimated that 7% of the over three trillion Google searches in 2024 – roughly 580 million per day – were health related^14–16^. (Fig. 1B)

## Declining Trust in the Healthcare System

**Fig. 2:**
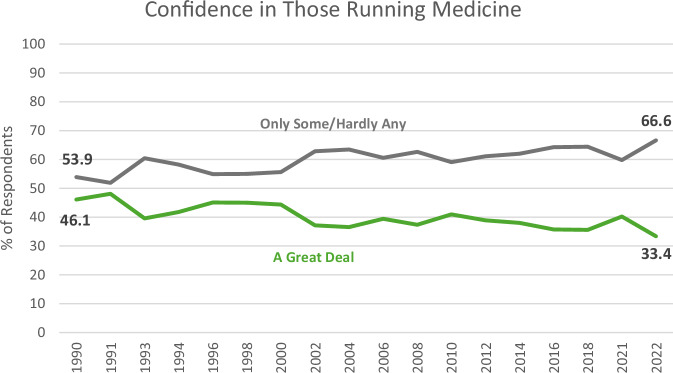
Confidence in the healthcare system from 1990 through 2022. Responses to “As far as the people running [medicine] are concerned, would you say you have a great deal of confidence, only some confidence, or hardly any confidence at all in them?”. (Data from reference #16).

Coincident with the explosive increase in internet use during the roughly two and a half decades of the Web 2.0 era, public trust and confidence in the US healthcare system has steadily declined. The General Social Survey—a long-running national study that tracks societal trends and public opinion—has consistently recorded this erosion of trust. One of its surveys, which measures confidence in institutions and their leadership, shows a notable drop in confidence in those running the healthcare system: from 1990 to 2022, the percentage of people expressing a “great deal” of confidence fell by approximately 13 percentage points, landing at just 33%^17^. This means that two-thirds of Americans now report having only “some” or “hardly any” confidence in healthcare leadership. (Fig. 2) At the same time, the proportion of people who believe that the US healthcare system is either in crisis or facing major problems has remained alarmingly high - hovering around 70% since 2000^18^.

## Factors Common to Internet Use and Healthcare Trust

While impossible to ascribe any simple explanation to this decrease in trust of the healthcare system, there are several issues that the online availability of health information provides a solution.

Public trust of US healthcare as an institution is based on how well it addresses the issues most important to the public^19^. In almost every year since 2000, these two issues have been cost and access^20^. In one survey, respondents put healthcare access and affordability in their top three public health concerns more often than addressing the opioid epidemic and preventing childhood deaths from gun violence^21^.

The internet, on the other hand, is an accessible information resource that costs only as much as the device to use it and a cellular or WiFi plan^6^. Even then, public libraries and public WiFi offer free alternatives to those costs. Therefore, while US healthcare costs and accessibility remain a continuing challenge, further eroding trust in healthcare institutions, the internet becomes an increasingly appealing alternative for a growing number of peoples’ health needs.

When it comes to trust in a personal healthcare provider, as opposed to the healthcare institution, interpersonal skills dominate^1,22^. One survey found that at least 75% of patients who did not trust their doctor cited reasons that were unrelated to medical knowledge or advice given^23^. Instead, they gave reasons such as “They spend too little time with me” (25%), “They do not know me” (14%), “They do not listen to me” (14%), and “They do not make eye contact with me” (3%)^23^. Research has shown that patients often decide to seek care elsewhere based on these socioemotional behaviors rather than perception of physician competence^24^.

On the other hand, the vast array of different perspectives and communities available online can provide individuals with the sense of a personal connection not experienced by some in traditional healthcare settings. Frequently, different personalities or influencers act as healthcare intermediaries who offer misinformation disguised as a simple, reassuring solution to an anxious individual with many questions about their health^25,26^. Often times, this is accompanied by an “us vs. them” rhetoric that encourages individuals to discredit those who disagree with them, like healthcare professionals^25–27^. This builds a sense of community that comforts the patient and fulfills the socioemotional connection they crave in a traditional healthcare provider, but often while driving them away from these providers^27^. The result is a cycle of distrust between patient and healthcare system underlain by valid feelings and concerns, but amplified by rhetoric and misinformation.

## Artificial Intelligence and the Potential to Regain Trust

Today, we are entering the era of Web 3.0, defined by greater personalization and user empowerment driven by breakthroughs in technologies like artificial intelligence (AI) and blockchain^3,9^. Among these, generative AI has been especially transformational, becoming one of the fastest adopted technological developments in history. ChatGPT, the first large language model (LLM) interface accessible to the general public, reached 100 million users in just two months, a milestone it took the internet 5 years to achieve^28^. A survey conducted in early 2025 revealed that half the population now use LLMs, with adoption rates showing little variation across education levels or socioeconomic status^28^.

Unlike traditional internet searches that often surface sponsored content and/or the loudest headlines, LLMs can deliver nuanced, context-rich responses distilled from millions of documents authored by medical professionals and other experts^29^. It would be important to explicitly note, however, that this very capacity for producing fluent, nuanced, and apparently credible text also introduces a specific risk: such outputs can constitute particularly compelling forms of disinformation. As shown by recent work, high epistemic plausibility and narrative coherence can substantially increase persuasive power, even when claims are misleading or incorrect^30,31^. This shift replaces oversimplified generalized results with access to a curated and nuanced summary of a subset of humanity’s collective knowledge, empowering individuals to make more informed decisions^29^. Thus, of the three factors discussed that make online information an appealing alternative to traditional healthcare (affordability, accessibility, and interpersonal validation), AI is at worst equivalent to the internet in the first two and substantially more advanced in the latter. If these factors do underlie decreasing trust in traditional healthcare and drive people to seek alternatives, it is reasonable to hypothesize that increased availability of LLM-based health agents to the general public will drive an exponential growth in people turning to online health resources first and often^32^. So, the question becomes, what is the best way to implement LLMs in medicine – for both patients and clinicians – in order to potentially grow patient trust in healthcare systems?

### Supporting Clinicians to Improve Patients’ Healthcare Experience

LLMs have the potential to significantly enhance the patient experience during healthcare encounters by alleviating the administrative burden placed on clinicians—particularly tasks related to electronic health records (EHRs)^28^. Presently, US physicians spend only 27% of their office time in direct interaction with patients, while nearly half (49%) is consumed by EHR and desk work^29^. Tasks such as assigning billing codes, writing prescriptions, and generating referrals are all examples of processes that AI can efficiently manage^31^. Moreover, AI-powered tools that transcribe patient encounters are already being adopted across many health systems, offering the potential to free up clinician time and foster more meaningful, uninterrupted interactions with patients^33–36^. In addition, information delivered both before and after a physician encounter can be customized to the patient’s past medical, social, and family history as well as be translated into a sixth-grade reading level, the accepted standard for health literacy, in the patient’s native language^33^.

Medical knowledge is rapidly expanding, patients are getting older with more comorbidities, medications, and specialists, and the exponential accumulation of EHR data means each patient is a “big data” challenge^37^. In many ways, medical complexity now exceeds the capacity of our minds^37^. Mistakes can cause misdiagnosis which can lead to burnout, financial instability, and death^37^. LLMs have begun to play a major role in addressing that. One example is OpenEvidence, a LLM specifically trained for medicine, that recently scored 100% on all three steps of the U.S. Medical Licensing Exam (USMLE) exams^38^. This platform also proved capable of providing evidence-based clinical decision support with high levels of physician satisfaction in a retrospective evaluation^39^.

### Supporting People’s Health Needs Outside the Clinical Setting

Considering the large number of people who already turn to the internet to answer their diagnostic health questions, the increasing availability of LLMs will substantially escalate and improve people’s potential to receive high-quality, individualized health information online. It is telling that one of OpenAI’s latest LLM version, GPT-5, is touted as their “best model yet for health-related questions” and that it “…acts more like an active thought partner, proactively flagging potential concerns and asking questions to give more helpful answers”^40^.

One example of how LLMs can serve as an always-available, expert health resource is a prospective, randomized, pragmatic study of Mo, a physician-supervised LLM-based conversational agent developed to provide medical advice^27^. The study found that relative to standard physician interaction, Mo was evaluated by patients to have higher levels of clarity, similar empathy and trust, and overall provided higher levels of satisfaction^41^. Another example is Articulate Medical Intelligence Explorer (AMIE), an LLM designed for diagnostic dialogue. In an evaluation involving simulated case scenarios with 159 patient actors, AMIE displayed superior accuracy and performance than primary care providers on 30 of 32 evaluation axes as judged by blinded medical specialists^42^. Similarly, Microsoft AI Diagnostic Orchestrator (MAI-DxO) was given 304 diagnostically challenging cases and achieved 80% accuracy - 4 times greater than the average of generalist physicians^43^. It also did so at 20% lower diagnostic cost^43^.

Another compelling recent example is the development of a multi-LLM-powered Personal Health Agent (PHA), designed to deliver personalized wellness recommendations by integrating multimodal data from consumer health devices and personal medical records^44^. The system comprises three specialized sub-agents—a data science expert, a health domain specialist, and a health coach—whose individual capabilities and collective performance were rigorously evaluated across 10 benchmark tasks. These assessments included over 1,100 evaluations from both end-users and healthcare professionals. The findings demonstrate that AI-driven agents can effectively analyze and interpret complex personal health data, and more importantly, offer empathetic, actionable guidance tailored to individual wellness needs.

Given the remarkable progress generative AI tools have already made in addressing a wide range of health needs, it’s crucial to recognize how early we still are in the development of this technology. ChatGPT, for example, has only been publicly available for ~three years. As these tools continue to evolve and as current challenges like bias, hallucinations, privacy, safety, liability, and regulatory concerns are systematically addressed, their appeal as accessible and affordable healthcare solutions will only grow. If healthcare systems fail to embrace LLMs as a collaborative partner, they risk being outpaced and outperformed by technology companies focused primarily on capitalizing on the desires of consumers, further eroding people’s trust in traditional healthcare systems.

## Conclusion

The adoption of AI, particularly LLMs, in healthcare is rapidly accelerating – likely, but not assuredly, for the better - because it can address many of the frustrations patients and providers face with current systems of care. Yet we remain at the earliest stages of determining how and where this transformative technology can be most effectively applied to fulfill the goal that “all peoples attain the highest possible level of health”^45,46^. There is still much work ahead, and the ultimate impact of LLMs will depend on where we focus our efforts and how well physicians and health system leaders understand both its promise and its risks. Like the internet before it, LLMs will reshape the landscape of medicine. The question is no longer whether to engage, but how. If clinicians and health systems do not take an active role in guiding their integration, they risk surrendering influence to less qualified voices - undermining both trust and the quality of care. The time has come for medicine not just to adopt LLMs, but to lead its responsible, patient-centered implementation.

## Data Availability

Data sharing is not applicable to this article as no datasets were generated or analyzed during the current study.
